# Supplementary material for: Learning curve and surgical outcome of robotic assisted colorectal surgery with ERAS program
Source: Sci Rep. 2022 Nov 29;12:20566. doi: 10.1038/s41598-022-24665-w (PMC9709162; doi:10.1038/s41598-022-24665-w)
Supplement: Supplementary file 1 — Supplementary Information 1. [file 41598_2022_24665_MOESM1_ESM.zip › LSC Raw data-Ñ[▒K/128/H_ñw╝╨░O▒Kñσ.pdf]

正式摘要(電子簽章：000000220017239)		
W52 / W52 ☐ 英文標題(注意:無英文姓名-請補病人基本檔!)	☐【讀取】憑證	

臺中榮民總醫院

出院病歷摘要

轉入號 : 轉出號 :

[redacted]

**(8)**流水編號	
**(9)**入院日期	110/05/26 (2021) 胃腸肝膽科 W52 - 145
**(10)**轉科日期	110/06/05 (2021) - 直腸外科
**(11)**出院日期	110/07/03 (2021) 住院天數計 38 日

診斷

- 入院 :1. LLQ abdominal pain, r/o appendicitis s/p Cefazolin(5/26~) + metronidazole(5/26~)
  2. Left femoral bone fr. s/p ORIF in 2018 at 澄清H.
  3. Hypertension for 10years without control.
- 出院 :1. FAP syndrome, with tubulovillous adenomas with focal high-grade dysplasia, pT1N0, s/p total colectomy + IRA on 2021/06/07, c/w anastomosis leakage
  2. Left femoral bone fr. s/p ORIF in 2018 at 澄清H.
  3. Hypertension for 10years without control.

主訴

- Acute onset of bilateral lower abdomen and flank pain (L't>R't) for 3-4 days.

病史

- This 30-year-old man with history of
  (1) Left femoral bone fr. s/p ORIF in 2018 at 澄清H.
  (2) Hypertension for 10years without control.
  This time he suffered from left lower abdomen and flank pain (L't>R't) for 3-4 days. He visited our ED on 5/25 where Lab datta showed no obvious abnormalities. KUB and L-spine x ray revealed no obvious lesion. Left flank stabbing pain was complained and improved after medication.Due to relative stable condition , he is discharged and OPD follow up. However, bilateral lower abdomen (L't>R't) got wrose with stool passage((病人不知道有沒有腹瀉)for 3 times on 5/25, so he came to our hospital again for help. Denied of fever, chills, upper respiratory tract symptoms, dysuria.TOCC(-)
  At our emergent department where initial vital sign showed BP:217/122mmHg, RR:18bpm, PR:94 bpm, BT:37.3℃. Physical examination showed alert and oriented consciousness, smooth respiratory pattern, abdomen mild distension with normal active bowel sounds, tenderness over A4,5,6 without rebounding pain and bilateral CV angle knocking pain , no pitting edema. Laboratory data showed unremarkable finding. The abdominal CT showed enlargement of appendix, about 1.1cm in short axis, early stage of appendicitis can't be R/O.Under the tentative diagnosed of lower abdominal pain, adequate IV hydration and empiric antibiotic with cefazolin and metronidazole were given. he was admitted for further management and treatment.

體檢發現

-
  - General appearance: A well-nourished person, with acute ill looking. Consciousness: Alert Pupil size: 2.0/2.0 mm Light reflex:
    - Normal light reflex

    Vital signs:

date	體溫(BT)	脈搏(P)	呼吸(R)	血壓(BP)	身高(BH)	體重(BW)	BMI
20210526	35℃	65次/分	17次/分	168/100mmHg	181.4Cm	125.6Kg	38.2
  - Skin
    - Normal skin turgor , No jaundice , No cyanosis , No rash , No wound / ulcer / scar
  - Lymphadenopathy
    - No lymphadenopathy
  - HEENT
    - Head: No shape deformity , No hair change , Normal lip
    - Eyes: ＊Conjunctiva: Normal , ＊Sclera: Not icteric , ＊Cornea: Clear , ＊Eyelids: Normal
    - Ears: No deformity
    - Nose: Normal appearance
    - Mouth and throat: No oral mucosa change , No throat injection
  - Neck
    - No thyroid enlargement , Neck: Jugular vein
  - Chest

-
    - Inspection: Normal and symmetric respiratory movement , No deformity , Normal breast
    - Palpation: No palpable mass , No tender point
    - Auscultation: No rales , No rhonchi , No wheezing , Chest / Auscultation / Stridor No stridor , No peripheral brochial sound , No friction rub
- Heart
    - Inspection: No visible apical impulse
    - Palpation: Not palpable PMI , No thrill , No heave
    - Auscultation: Regular heart beats , No S3 , No S4 , No murmur , No pericardial friction rub
- Abdomen
    - Inspection: Distension - + , No striae
    - Auscultation: Normal bowel sound ,
    - Palpation: No hepatomegaly , location: A4,5,6 , No rebounding pain , No palpable mass , No splenomegaly
    - Percussion: No shifting dullness
- Genito-anus patient refuse
- Back and spine
    - No deformity , No knocking pain
- Extremities
    - No deformity , No clubbing finger , No pitting edema , Normal joint , Normal peripheral pulse
- Neurological examination
    - No mental status change
    - Normal cranial nerve
    - Normal meninges
    - Normal motor
    - Normal sensory
    - Normal cerebellum
    - Normal Stance & Gait

手術日期及方法

- 手術日期及方法20210607 1. Conventional total colectomy with side to end, ileorectal anastomosis (IRA)
  # Initially, laparocopic was attempted. It was later shifted to conventional, opening method.
  2. Intraoperative colonoscopy : (-)
  3. High ligation (+) : At IMA root. IMV and left colic artery were also ligated. Ileocolic vessels were also ligated. Distal middle colic vessels were ligated.
  4. EndoGIA (60-4.1) X3 + EndoGIA (60-3.5) X1 + EEA (25 mm) were applied.
  5. One 12 Fr. penrose drainge tube was placed from LLQ of abdomen and inserted into the pelvic area
  6. The colon was taken down from the splenic flexure site and hepatic flexure site

住院治療經過

- After admisison, general surgeon on duty was consulted twice which appendicitis was less likely according to clinical presentation. The pain improved after NPO and antibiotic treatment. Acute onset of bloody tinged stool on 5/30. The sigmoid scope on the next day revealed R-S-D colon numerous polyps, suspect adenomatous polyposis syndrome. Painless esophagogastroduodenoscopy showed multiple polyps over lower, middle, high body and fundus,susp. fundic gland polyps, s/p Bx, and colonscope showed numerous polyps with several huge sized polyps. We consult CRS for suspect amilial adenomatous polyposis. Total colectomy + IRA were done on 2021/06/07 smoothly. Neverheless, nausea and vomiting were noted on 06/08 which NG decompression of 3100ml was noted. He self removed the NG today which was re-on later due to abdominal distension. Turbid drain was noted on 06/15 suspect leakage. We shifted Abx to Tapimycina and add TPN. His abdominal pain and ileus improved gradually. Diarrhea was complainted after improved oral feeding, improved after Loperamide use. Penrose drain was retracted gradually, and finally removed on 7/02. Under a relative stable condition, the patient was discharged with OPD follow up.

合併症

一般檢查

- 累積報告 :

DATE	TIME	ALB	TP	BIL.T	BIL.D	ALKP	AST	ALT	LDH	AMY	LIPASE	NH3	Ferritin	UA
1100525	0839			0.5				39						
1100525	2350										41			
1100528	0841	4.4												
1100607	1830			0.5				44						
1100609	0854			0.45				22	260					
1100615	0904			0.29				47						
1100617	0840	3.5		0.36		128		88						
1100621	0843	3.8												
1100628	1040	4.2		0.46	0.15		52	98						

DATE	TIME	WBC	RBC	HGB	HCT	MCV	PLT	BLST	NEUT	BAND	SEG	LYM	MON	EOS	BAS

1100525	0834	10650		14.1			334		66.0			23.6	7.3	2.3	0.8
1100528	0853	8990		12.7			301		70.9			17.4	6.8	3.9	1.0
1100607	1829	18930		13.1	40.8		282		93.4			3.3	3.0	0.1	0.2
1100609	0843	14150		12.5	39.1		268		84.3			7.7	7.1	0.5	0.4
1100614	1256			11.6	36.4										
1100615	0909	18440	4.28	11.5	34.9	81.5	349		85.5			7.4	4.8	1.9	0.4
1100617	0848	16910		11.5	34.4		377		85.0			8.1	4.5	2.2	0.2
1100621	0927	10700		11.8					75.5			12.7	6.5	4.6	0.7
1100628	1039	5910		12.7	39.5		380		62.6			17.8	11.2	6.9	1.5

DATE	TIME	NA	K	CL	BUN	CREAT	eGFR	CA	Mg	P	CRP	ESR	PCT	D-Dimer	LACTATE
1100525	0839	138	3.8	103	6	1.08	85.33	9.7			0.021				
1100525	2349													0.24	
1100528	0841	142	3.8			1.05	88.15	9.2							
1100607	1830	139	4.0		7	0.98	95.45	8.7							41.1
1100609	0848														11.3
1100609	0854	139	4.0		4	0.89	106.67				2.796				
1100615	0904	141	3.9		9	0.90	105.31	8.4							
1100617	0806														4.9
1100617	0840	138	3.8		5	0.98	95.45	8.7	2.0	3.6	1.052				
1100621	0843	139	4.1					9.1			0.468				
1100628	1040		4.2	102	8	1.04		9.7							

DATE	TIME	GLUCOSE	HbA1C	CHOL	TG	HDL	LDL	KETONE	CK	CKMB	TRO-T	PT-P	APTT-P	NT-pro BNP
1100525	0834											10.7	27.3	
1100525	0839	87												
1100528	0841		5.7											
1100606	0030											10.7	27.1	
1100617	0840			128	91									
1100617	0849	107												

DATE	TIME	SP-GR	PH	PROT	GLUC	KETON	BILI	UROB	OB	NITR	LEU	RBC	WBC	EPCEL
1100525	0949	1.007	6.0	Negative	Negative	Trace	Negative	0.2	Negative	Negative	Negative	0-2	0-2	0-1
1100601	1526	1.005	6.0	Negative	Negative	Negative	Negative	0.2	Negative	Negative	Negative	0-2	0-2	0-1

DATE	TIME	STOOL-OB	FAT	RBC	WBC	PARA
1100527	0633			-	-	Not Found
1100530	1330	4+				

DATE	TIME	PH	BEB	SCVO2	PO2	HCO3	HGB	PCO2	TCO2
1100525	0834	7.343	-3.5	79.6	47.2	22.1		41.6	23.4
1100607	1829	7.351	-4.6	99.5	186.6	20.5		37.9	21.6
1100609	0855	7.332	1.0	50.1	29.2	28.0		54.1	29.7

特殊檢查

病理報告

- 活體組織切片病理報告　手術日期：**110/06/07**　病理號：**S1018512**

診斷醫師:李欣倪 報告日期:110/06/15
Pathologic diagnosis:
Colon, terminal ileum, and appendix, total colectomy --- Staus post-polypectomy. Multiple tubular adenomas and tubulovillous adenomas with focal high grade dysplasia, involving the whole colorectum. The appendix, ileocecal valve, bilateral cut ends and mesocolic lymph nodes (0/77) are free of malignancy.
Ancillary study for diagnosis:
Deep cut done on section F.
Prognostic and predictive factor:
Combined S1018243
1. Tumor size: 0.8 cm.
2. Depth of invasion: Submucosa.
3. Histologic grade: G2: Moderately differentiated.
4. Angiolymphatic invasion: Not identified.
5. Perineural invasion: Not identified.
6. Tumor deposits (discontinuous extramural extension): Not identified.
7. Macroscopic tumor perforation: Not identified.
8. Surgical Margins: a) Proximal Margin: Uninvolved by invasive carcinoma. b) Distal Margin: Uninvolved by invasive carcinoma. c) Radial or Mesenteric Margin: Uninvolved by invasive carcinoma.
9. Lymph node status: Uninvolved (0/77).
10. Extranodal involvement: Not identified.
11. Tumor regression grading S/P CCRT: No known presurgical therapy.
12. Type of polyp in which invasive carcinoma arose: Tubulovillous adenoma.

- 13. Additional pathologic findings: Adenoma(s).
  14. Pathological TNM Stage: pT1N0(According to the eighth edition, American Joint Committee on Cancer Staging Guidelines for Tumors).
  15. TNM descriptors: Not applicable.
  Gross description:
  The specimen consists of a totally resected colon with anus, 73 cm in length and 7 cm in distal circumference, terminal ileum, 11 cm in length and 5 cm in circumference, an unremarkable appendix, 5x1x1 cm in length, omentum, 25x10x1 cm, and mesocolic and perirectal soft tissue. Three are two pedunculated polypoid tumors and one gray tan firm flat tumor, 2x1.5x1 cm (tumor 1) and 1x0.5x0.5 cm (tumor 2) and 1.5x1x0.5 cm (tumor 3), are noted, 10 cm, 41 cm and 55 cm in distance to the distal cut end, respectively. There are also numerous polyps over the whole colorectal mucosa. The both cut ends are free of tumor or polyps grossly. Representative part for section: A) colon cut end B) small intestine cut end C) ileocecal valve D) appendix E-F) tumor 1 G) tumor 2 H-J) tumor 3 K-T) numerous polyps over colon U) small intestine V-AB) lymph nodes.
  #T-59420_2 #M-82110_2 0 1105 000000
  #T-C4560_2 #M-09183_2 0 1105 000000
  #T-59200_2 #M-09183_2 0 1105 000000

其它

出院時情況

- 3.改門診治療

出院指示

藥名	劑量	劑量單位	用法	頻次	數量	數量單位	現況	天數	單位
Otilonium tab 40mg	1	TAB	PO	TID	36	TAB	TH	12	天
Econ tab 40mg	1	TAB	PO	QID	48	TAB	TH	12	天
5/160mg Exforge tab	1	TAB	PO	QD	12	TAB	TH	12	天
Ulstop tab 20mg	1	TAB	PO	BID	24	TAB	TH	12	天
CefaDROxil-500 cap	1	CAP	PO	Q12H	14	CAP	TH	7	天
Metronidazole cap 250mg	2	CAP	PO	TIDPC	42	CAP	TH	7	天

出院計劃

- 門診日期
  - date on 2021/07/13
- Closely monitor left abdominal wound after penrose drain removed.

主治醫師 2062F 蔣鋒帆 / 住院醫師 2345B 陳映晴

正式摘要(電子簽章：000000220017239)

W52 / W52

doctorRank:B
